# Draft Genome Sequence of *Arcticibacter svalbardensis* Strain MN12-7^T^, a Member of the Family *Sphingobacteriaceae* Isolated from an Arctic Soil Sample

**DOI:** 10.1128/genomeA.00484-13

**Published:** 2013-07-11

**Authors:** S. Shivaji, Sreenivas Ara, Sathish Prasad, B. Poorna Manasa, Zareena Begum, Aditya Singh, Anil Kumar Pinnaka

**Affiliations:** CSIR–Centre for Cellular and Molecular Biology Habsiguda, Hyderabad, Andhra Pradesh, Indiaa; CSIR–Institute of Microbial Technology, Sector 39A, Chandigarh, Indiab

## Abstract

The 4.69-Mb genome sequence of *Arcticibacter svalbardensis* strain MN12-7^T^, isolated from an Arctic soil sample, is reported.

## GENOME ANNOUNCEMENT

*Arcticibacter* *svalbardensis* strain MN12-7^T^ is a Gram-negative, rod-shaped, pigmented, nonmotile bacterium isolated from a surface soil sample from Ny-Ålesund, Svalbard, the Arctic (1). The closest phylogenetic neighbors of *Arcticibacter svalbardensis* strain MN12-7^T^, based on the 16S rRNA similarity, were found to be *Pedobacter tournemirensis* strain TF-37.2-LB10^T^ (93.18%), *Pedobacter xinjiangensis* strain 1257^T^ (93.02), and *Mucilaginibacter lutimaris* strain BR-3^T^ (92.49%).

The genome of *Arcticibacter svalbardensis* strain MN12-7^T^ was sequenced using the Roche 454 (FLX titanium) pyrosequencing platform. The sequencing yielded 111,666,211 bases in 321,116 reads, which is a 23× coverage of the genome. The assembly of the raw sequencing data was performed using GS De Novo Assembler (version 2.8; F. Hoffmann-La Roche, Ltd., Switzerland). Assembly yielded 151 contigs. All the contigs were ≥546 bp, with the largest contig 170,506 bp. A total of 4,659,080 bp (99.36%) were above the quality score of 40, with an average quality score of 63.69. The G+C content of DNA of *Arcticibacter svalbardensis* strain MN12-7^T^ was calculated to be 38.16 mol%. The genome annotation was performed using RAST (Rapid Annotations using Subsystems Technology) (2), tRNA was predicted by tRNAscan-SE (v. 1.23) (3), and rRNA genes were predicted by RNAmmer (v. 1.2) (4).

The annotation has 4,356 predicted genes, including three 5S rRNA, one 23S rRNA, one 16S rRNA, and 40 tRNA genes. In the annotation there are 65 genes encoding resistance against antibiotics and toxic compounds, including 4 genes for arsenic resistance, 2 genes for mercuric resistance, 2 genes for zinc resistance, 19 genes for cobalt-zinc-cadmium resistance, and 6 genes for copper homeostasis. Seventy-four stress response genes were also predicted in the annotation. There are 101 genes for membrane transport, including 88 Ton and Tol transport systems.

The functional comparison of the genome sequence using the SEED Framework for Comparative Genomics (http://www.theseed.org) revealed the closest neighbors of *Arcticibacter svalbardensis* MN12-7^T^ as *Pedobacter heparinus* strain DSM 2366 (SEED Viewer identification no. 485917.5), *Sphingobacterium spiritivorum* strain ATCC 33300, *Pedobacter* sp. strain BAL39, *Sphingobacterium spiritivorum* strain ATCC 33861, and *Algoriphagus* sp. strain PR1, with similarity scores of 530, 503, 478, 465, and 254, respectively.

The sequencing of this Arctic bacterium will assist in increasing our understanding of evolution by gathering knowledge through the genome-level study of the less explored, extreme conditions of the Arctic region.

### Nucleotide sequence accession numbers.

The draft genome sequence of *Arcticibacter svalbardensis* strain MN12-7^T^ has been deposited in DDBJ/EMBL/GenBank under the accession number AQPN00000000. The version described in this paper is the first version, AQPN01000000.
